# Knowledge, attitudes, and practices of pediatricians in Crete, Greece, regarding RSV immunization: a cross-sectional study

**DOI:** 10.1007/s00431-026-06977-5

**Published:** 2026-04-27

**Authors:** D. Vrentzos, I. Pavlopoulou, G. A. Syrogiannopoulos, I. Grivea, F. Ladomenou

**Affiliations:** 1 Department of Pediatrics, Panagioti & Aglaia’s Kyriakou Children’s Hospital, Athens, Greece; 2 Pediatric Research Laboratory, Faculty of Nursing, National and Kapodistrian University of Athens, Athens, Greece; 3 Department of Paediatrics, Faculty of Medicine, School of Health Sciences, University of Thessaly, Larissa, 41500 Greece; 4 Department of Pediatrics, Faculty of Medicine, School of Health Sciences, University of Ioannina, Ioannina, 45500 Greece

**Keywords:** Respiratory syncytial virus (RSV), Pediatricians, Knowledge, attitudes, and practices (KAP), Immunoprophylaxis, National immunization programs

## Abstract

Respiratory syncytial virus (RSV) is a leading cause of severe lower respiratory tract infections in early infancy, and the recent introduction of two novel immunoprophylaxis tools—maternal vaccination with the RSVpreF vaccine and infant protection with the long-acting monoclonal antibody nirsevimab—represents a major advancement in RSV prevention. The successful implementation of these strategies depends heavily on pediatricians’ awareness, confidence, and readiness to integrate them into routine clinical practice. Evidence from Southern Europe remains limited. This cross-sectional study assessed the knowledge, attitudes, and practices of pediatricians in Crete, Greece, regarding RSV infection and emerging prevention tools. A structured electronic questionnaire was distributed between April and July 2025 to all board-certified pediatricians and pediatric residents registered with the regional medical associations. Among 169 respondents (mean age 46 ± 12.5 years; 83.4% women), overall knowledge was high, with a mean score of 89.6 ± 10.0. Knowledge gaps were concentrated in areas related to newly introduced immunoprophylaxis tools, including awareness of RSV monoclonal antibody availability for infants, updated recommendations covering infants up to 6 months, and distinctions between palivizumab and nirsevimab. Attitudes toward vaccination were overwhelmingly positive: nearly all participants expressed strong vaccine confidence and adherence to national immunization guidelines. Most pediatricians (78.6%) felt adequately informed about RSV. These findings suggest that pediatricians in Crete possess a strong foundation for integrating new RSV prevention strategies, although targeted educational initiatives—particularly regarding nirsevimab—will be essential to ensure consistent and equitable implementation. The results provide timely insights to support national planning and optimize the rollout of RSV immunoprophylaxis in Greece and comparable settings.

**Table:** 

**What is Known:** • *RSV is a leading cause of severe respiratory disease in early infancy.* • *New prevention tools (RSVpreF, nirsevimab) are being introduced into national programs.* • *Pediatricians’ knowledge and confidence strongly influence vaccine uptake.*	
**What is New:** • *Greek pediatricians show high overall RSV knowledge and strong vaccine confidence.* • *Specific gaps exist regarding nirsevimab and distinctions between immunoprophylaxis tools.* • *Findings highlight targeted education needs during early implementation of RSV prevention.*

**Supplementary Information:**

The online version contains supplementary material available at 10.1007/s00431-026-06977-5.

## Introduction

Respiratory syncytial virus (RSV) represents the primary cause of acute lower respiratory tract infections (LRTIs) in early infancy and is a major contributor to mortality among babies under 6 months of age [1]. Most hospitalizations due to severe RSV disease occur between 6 weeks and 6 months of life, creating a substantial strain on healthcare systems and underscoring the critical importance of effective preventive measures [2]. Recent estimates indicate that, among infants aged 0–6 months, RSV-related lower respiratory tract infections account for approximately 6.6 million cases and 1.4 million hospital admissions worldwide [3]. Despite the substantial disease burden during the first 6 months of life, no RSV vaccine is currently licensed for direct use in the pediatric population; protection in early infancy is achieved through maternal immunization or monoclonal antibody administration. For over 25 years, palivizumab, a monoclonal antibody targeting the F-glycoprotein of the virus, was the only prophylaxis of RSV-related lower respiratory tract infections (LRTIs), limited to high-risk infants due to monthly dosing and high costs.

Recently, two new immunization strategies have transformed RSV prevention. Nirsevimab, a long-acting monoclonal antibody administered as a single dose, was authorized by the European Medicines Agency (EMA) in October 2022 and has been shown in both clinical trials and real-world studies to provide season-long protection for all infants—regardless of risk status—by reducing RSV-related lower respiratory tract infections and associated hospitalizations by approximately 70–90% [4–6]. In parallel, seasonal administration of the bivalent recombinant stabilized prefusion F protein subunit vaccine (RSVpreF), which received approval from the ΕΜΑ in August 2023, enables transplacental antibody transfer providing protection from birth during the highest-risk months of life and is available for pregnant women between 32 and 36 weeks of gestation to enhance neonatal protection through transplacental antibody transfer [7]. Both clinical trials and real-world data have demonstrated significant reductions in medically attended RSV LRTIs and hospitalizations among young infants, whose mothers were vaccinated during pregnancy [1, 8]. Following European authorization, RSVpreF was incorporated into the Greek National Immunization Program (NIP) in January 2025, and nirsevimab was subsequently introduced in October 2025 [9, 10]. Together, these approaches represent a major shift toward broader, more practical, and more equitable RSV prevention.

However, the successful implementation of these new preventive strategies relies heavily on pediatricians’ awareness, acceptance, and readiness to integrate them into routine clinical practice. International evidence consistently demonstrates that healthcare providers play a pivotal role in the uptake of both maternal and infant immunization programs, with studies from the United States, Canada, and several European countries showing that provider recommendation is one of the strongest determinants of parental acceptance [11, 12]. Despite this, limited evidence exists regarding pediatricians’ preparedness to implement RSVpreF and nirsevimab in real-world settings, particularly in Southern Europe, where data on provider familiarity with these newly introduced tools remain scarce. Moreover, gaps in provider knowledge, uncertainty regarding vaccine effectiveness or safety, and limited familiarity with monoclonal antibody programs have been associated with suboptimal implementation in early rollout evaluations [13]. Early assessments from other countries reveal considerable variability in provider awareness and confidence, underscoring the need for context-specific evaluations to guide national implementation strategies [14, 15]. Yet, despite the rapid introduction of these novel prevention tools, it remains essentially unknown to what extent pediatricians are adequately informed, prepared, and willing to incorporate them into everyday clinical practice.

The primary objective of this study is to assess the knowledge, attitudes, and practices of pediatricians in Crete regarding RSV infection. A secondary aim is to evaluate their familiarity with both established and newly introduced immunization tools against the virus.

## Materials and methods

### Study design and setting

This was a cross-sectional Knowledge, Attitudes, and Practices (KAP) study conducted among pediatricians practicing on the island of Crete, the largest island in Greece, with a population of approximately 630,000 residents distributed across four regional units (Heraklion, Chania, Rethymno, and Lasithi). Healthcare services are delivered through a mixed public–private system. The target population included board-certified pediatricians and pediatric residents employed in primary, secondary, and tertiary healthcare facilities, as well as those working in private practice. Data collection took place between April and July 2025, shortly after the introduction of RSVpreF into the National Immunization Program (January 2025) and prior to the incorporation of nirsevimab (October 2025).

### Questionnaire development and validation

The questionnaire was developed specifically for this study following a targeted review of the literature on RSV epidemiology, immunoprophylaxis, and KAP survey methodology. Item generation was guided by the study objectives and by current national and international recommendations on RSV prevention. The full questionnaire is provided in Appendix A. Attitude items assessed confidence in vaccines, adherence to national recommendations, and perceived adequacy of information. Practice items assessed self-reported adherence to immunization guidelines and routine counseling behaviors. These domains were analyzed separately.

### Content validity

Two senior pediatricians with expertise in pediatric infectious diseases independently reviewed all questionnaire items for relevance, accuracy, and alignment with contemporary RSV prevention guidelines. Their feedback resulted in refinement of item wording, removal of ambiguous or redundant questions, and improved correspondence between knowledge items and current immunoprophylaxis strategies.

### Face validity

A pilot test was conducted with 10 pediatricians who were not part of the final study sample. Participants evaluated the clarity of instructions, comprehensibility of items, and overall flow of the questionnaire. Minor linguistic and structural adjustments were made based on their comments, including simplification of phrasing and reordering of selected items to enhance readability.

Internal consistency: Internal consistency was assessed using Cronbach’s alpha for the knowledge section. This section consisted of 15 dichotomous (true/false) items, all of which were included in the calculation, covering RSV epidemiology, clinical burden, and available immunoprophylaxis tools (Appendix A). The resulting Cronbach’s alpha of 0.762 indicates acceptable internal consistency for a newly developed instrument with heterogeneous content. Attitude and practice items were analyzed individually, as they were not designed to form a unified scale.

#### Language

The questionnaire was administered in Greek, the native language of all participants.

### Sampling and recruitment

All actively practicing pediatricians and pediatric residents registered with the Medical Associations of the four regional units of Crete were eligible to participate, yielding a total sampling frame of 325 individuals. The survey was distributed electronically via Google Forms through the regional Medical Associations. Participation was voluntary and anonymous. A total of 169 pediatricians completed the questionnaire, corresponding to a response rate of 52% (169/325).

### Sample size and power analysis

**Fig. 1:**
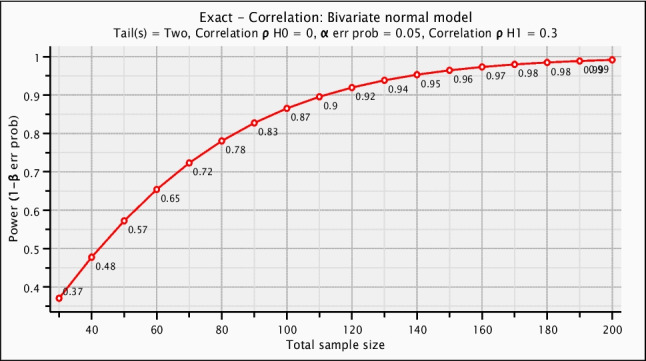
Sample size determination via power analysis

The study relied on voluntary participation, resulting in a self-selected, non-probability sample. An a priori power analysis was performed to determine the minimum number of participants required to detect a true association, if present. An initial (uncorrected) G*Power calculation indicated that 146 participants would be required to detect a medium effect size (*r* = 0.30) with 95% power at *α* = 0.05. After applying the finite population correction for the total population of 325 pediatricians, the required sample size was reduced to 138 (Fig. 1). The modest reduction after applying the finite population correction is expected, as the uncorrected sample size already represented a substantial proportion of the total population. A higher power level (95%) was selected due to the small, finite size of the target population and the anticipated subgroup comparisons (e.g., specialists vs. residents), in order to minimize the risk of Type II error and ensure adequate sensitivity. The final sample (*n* = 169) exceeded the minimum required number.

### Statistical analysis

Descriptive statistics were used to summarize the data. Continuous variables were expressed as mean ± standard deviation, whereas categorical variables were presented as absolute and relative frequencies. Normality of continuous variables was assessed using visual inspection of histograms and Q–Q plots. Associations between a continuous variable and a dichotomous variable were examined using the point-biserial correlation coefficient. Relationships between two continuous variables were assessed using Pearson’s correlation coefficient. Given the sample size and distributional characteristics, non-parametric tests were considered; however, parametric tests were retained due to acceptable normality patterns and to facilitate interpretability. The threshold for statistical significance was set at 5%. All analyses were performed using SPSS version 27 (IBM Corp., Armonk, NY).

### Ethical considerations

The study was approved by the Ethics Committee of the 7th Health Region of Greece and conducted in accordance with the principles of the Declaration of Helsinki. All participants were informed about the purpose and procedures of the study prior to their involvement. Participation was entirely voluntary, and respondents were assured that their answers would remain anonymous and confidential. Completion and submission of the questionnaire were considered to constitute informed consent.

## Results

A total of 169 pediatricians completed the survey, corresponding to a response rate of 52% (169/325). Participants had a mean age of 46 ± 12.5 years, and the majority were women (*n* = 141, 83.4%). Most respondents were board-certified pediatricians (*n* = 136, 80.5%), while 33 (19.5%) were pediatric residents.

### Knowledge assessment

The mean overall knowledge score was 89.6 ± 10.0 percentage points, reflecting a high level of understanding of RSV epidemiology, clinical burden, and available immunization strategies. As shown in Table 1, performance across individual knowledge items was consistently high. When stratified by age group, biological sex, and professional status, median scores remained above 87/100 in all subgroups, indicating uniformly strong knowledge across the sample.

**Table 1:** Distribution of correct and incorrect responses for each item

	Wrong answer		Right answer		Correct answer
	*Ν*	*N* %	*Ν*	*N* %	
Is there currently an available and safe RSV vaccine for the pediatric population?	57	33.7%	112	66.3%	False
Do the most recent RSV immunization guidelines include infants up to 6 months of age?	42	24.9%	127	75.1%	True
Do the most recent RSV immunization guidelines include children and adolescents?	9	5.3%	160	94.7%	False
Do the Greek national guidelines provide specific recommendations regarding maternal RSV immunization during pregnancy?	21	12.4%	148	87.6%	True
Is RSV vaccination recommended for pregnant women according to the latest guidance of the National Immunization Committee?	18	10.7%	151	89.3%	True
Can RSV be transmitted through food consumption?	14	8.3%	155	91.7%	False
Is RSV transmitted via respiratory droplets?	2	1.2%	167	98.8%	True
Does RSV share common clinical symptoms with influenza?	3	1.8%	166	98.2%	True
Is a specific antiviral treatment available for RSV infection?	22	13.0%	147	87.0%	False
In Europe, is the RSV epidemiological season recognized as occurring from November to March?	5	3.0%	164	97.0%	True
Are ready-to-use monoclonal antibodies available for the immunoprophylaxis of newborns and infants?	7	4.1%	162	95.9%	True
Are there differences between the two monoclonal antibodies used for RSV prevention, Synagis (palivizumab) and Beyfortus (nirsevimab)?	38	22.5%	131	77.5%	True
Is Synagis (palivizumab) administered to newborns and infants with specific underlying medical conditions?	21	12.4%	148	87.6%	True
Is apnea one of the most common complications of severe RSV infection in infants under one year of age?	11	6.5%	158	93.5%	True
Are antibiotics effective in the treatment of RSV infection?	1	0.6%	168	99.4%	False
Do maternal RSV antibodies reduce the risk of infection in infants during the first 4–6 months of life?	10	5.9%	159	94.1%	True

*N* number of participants, *N*% percentage of participant

The three items with the lowest proportion of correct responses were:

1. Awareness that no RSV vaccine is currently licensed for pediatric use (33.7% incorrect),
2. Understanding that recent RSV immunization recommendations include infants up to 6 months of age (24.9% incorrect), and
3. Knowledge of differences between the two available monoclonal antibodies, palivizumab and nirsevimab (22.5% incorrect).

Correlation analyses were performed at both the item level (for the three knowledge items with the lowest proportion of correct responses) and the category level (overall knowledge score). Among the three individual items, only the first—awareness that no RSV vaccine is currently licensed for pediatric use—showed a statistically significant association with professional status. Among residents, the proportion of correct responses was 51.5% (95% CI: 34.4–68.6%), whereas among specialists it was 69.9% (95% CI: 59.4–80.4%). The difference between the two groups was statistically significant (*χ*^2^(1) = 3.995, *p* = 0.043).

No significant associations were observed between the overall knowledge score and age (*p* = 0.156), biological sex (*p* = 0.562), or professional status (*p* = 0.053). *p*-values were not adjusted for multiple testing**,** as the analyses were exploratory and limited in number, and applying strict corrections (e.g., Bonferroni) would substantially increase the risk of Type II error in this context.

A summary of the item-level correlation analyses is presented in Table 2.

**Table 2:** Correlation analysis of knowledge items and participant characteristics

Knowledge item	Variable tested	Test	*p*-value	Significant
Awareness that no RSV vaccine is licensed for pediatric use	Professional status	*χ*^2^	0.043	Yes
Understanding that recommendations include infants up to 6 months	Professional status	*χ*^2^	> 0.05	No
Distinction between palivizumab and nirsevimab	Professional status	*χ*^2^	> 0.05	No
Overall knowledge score	Age	Pearson r	0.156	No
Overall knowledge score	Sex	*t*-test	0.562	No
Overall knowledge score	Professional status	*t*-test	0.053	No

### Attitudes and practices

In this study, attitude items reflected general vaccine confidence and perceptions of vaccine importance, whereas practice items referred to self-reported adherence to national immunization recommendations. Both attitudes and practices toward vaccination were overwhelmingly positive. A total of 163 participants (96.4%) reported adhering to National Immunization Committee recommendations for both personal and patient immunization. Nearly all respondents (168/169, 99.4%) expressed general confidence in vaccines. All participants agreed that vaccines are essential for preventing infectious diseases and protecting public health, and 168 (99.4%) believed that vaccines substantially reduce morbidity and mortality.

Regarding RSV-specific knowledge, 132 pediatricians (78.6%) reported feeling adequately informed about RSV infection, whereas 37 (21.4%) described their knowledge as moderate.

## Discussion

To our knowledge, this is the first study in Greece to focus exclusively on pediatricians and to evaluate their knowledge, attitudes, and practices regarding the full spectrum of emerging RSV prevention strategies. Previous Greek research included mixed groups of health professionals, with pediatricians representing only part of the sample, and did not specifically assess specialty-focused capacity within the context of rapidly evolving national RSV recommendations [16]. Addressing this gap, our findings indicate consistently high knowledge levels (mean score 89.6%), strong vaccine confidence, and broad adherence to national guidelines among pediatricians in Crete, while also identifying targeted gaps related to newly introduced RSV immunoprophylaxis tools. Notably, knowledge was uniformly high across age groups, biological sexes, and levels of training, suggesting a well-informed and cohesive pediatric workforce. Taken together, these findings suggest that Greek pediatricians possess a solid foundation for integrating emerging RSV prevention tools, while targeted education on nirsevimab will be essential to ensure consistent and equitable implementation.

The local context appears to play a meaningful role. Crete has historically reported some of the highest childhood vaccination coverage rates in Greece, supported by a well-established pediatric care network and long-standing continuity between families and their pediatricians [17]. This environment may help explain the notably strong performance observed in our sample and suggests that regions with robust vaccination cultures may be better positioned to incorporate new RSV preventive tools rapidly and consistently. The near-universal confidence in vaccines observed in our study (99.4%) further reinforces this favorable landscape, as high trust in immunization is a known facilitator of early adoption of new preventive interventions [18]. This “advantageous starting point” is an important insight for countries planning the rollout of nirsevimab or maternal RSV vaccination.

When placed within the international literature, the level of familiarity demonstrated by Greek pediatricians compares favorably. Studies from Italy and Turkey report substantial knowledge gaps regarding RSV transmission, maternal immunization, and monoclonal antibodies [19–21], while research from Croatia and the United States shows more variable awareness influenced by demographic and regional factors [22, 23]. Countries that implemented nirsevimab early—such as France, Spain, and the United States—demonstrate markedly higher clinician familiarity, underscoring the strong link between national implementation and provider awareness [15]. Our findings align with this pattern: although pediatricians demonstrated strong foundational knowledge of RSV epidemiology and clinical management, uncertainty remained specifically around nirsevimab indications and distinctions between available monoclonal antibodies. These were among the items with the lowest proportion of correct responses, reflecting the limited real-world use of nirsevimab in Greece at the time of data collection. Interestingly, only one knowledge item—regarding the existence of a pediatric RSV vaccine—differed significantly between board-certified pediatricians and residents, suggesting that gaps are concentrated in areas where recommendations have evolved most rapidly. This difference, although modest in magnitude, is statistically robust and consistent with the expected variation between clinicians at different stages of training. The comparison was based on a standard test of proportions, which is appropriate for dichotomous outcomes such as correct versus incorrect responses, supporting the validity of the observed association.

Although this study focuses on pediatricians, it is important to note that the two available RSV prevention strategies in Greece—maternal vaccination with RSVpreF and infant immunization with nirsevimab—are implemented by different provider groups. Pediatricians are responsible for nirsevimab administration, whereas obstetricians and gynecologists guide maternal vaccination. In Greece, childhood vaccination coverage is consistently high and antenatal care is well structured, yet maternal vaccine uptake remains disproportionately low, as highlighted in analyses of maternal immunization practices [24]. Low awareness of RSV and limited intention to receive RSV vaccination during pregnancy have also been documented among pregnant women in Greece [25], further underscoring that maternal RSV vaccination depends on determinants distinct from pediatric uptake. These differences indicate that the successful implementation of RSVpreF cannot be assumed to mirror pediatric patterns. A complementary assessment among obstetricians and gynecologists would therefore be valuable to provide a more complete picture of national readiness for RSV prevention.

This study presents several notable strengths. It was conducted within a clearly delineated population of pediatricians, allowing broad coverage of the target group and reducing uncertainty regarding the sampling frame. The questionnaire was developed through a systematic process that included expert review, pilot testing, and evaluation of internal consistency, thereby supporting the reliability of the instrument. Importantly, data collection occurred during a period of rapid evolution in RSV prevention policy, enabling the study to capture clinicians’ perspectives at a critical juncture in the introduction of new immunization approaches. The relatively high proportion of respondents from the eligible population further enhances confidence in the robustness of the dataset. Taken together, these elements provide timely insight into pediatricians’ ability to interpret and communicate emerging RSV immunization recommendations.

Several limitations should also be considered when interpreting these findings. Participation was voluntary, resulting in a self-selected, non-probability sample that may not fully represent the broader pediatric workforce. Respondents may differ from non-respondents in their engagement with immunization issues or familiarity with evolving RSV guidance, introducing the potential for selection bias. Moreover, because participants were aware that their knowledge and practices were being evaluated, a Hawthorne effect cannot be ruled out, as some may have provided responses more aligned with recommended practice than with their routine clinical behavior [26]. The cross-sectional design captures attitudes and practices at a single point in time and does not account for changes that may occur as RSV immunization policies continue to develop. Additionally, reliance on self-reported practices may introduce social desirability bias. These limitations underscore the need for future research using probability-based sampling and longitudinal designs to enhance generalizability and monitor changes over time.

Another limitation is the absence of previously validated instruments specifically designed to assess attitudes and practices related to the newly introduced RSV immunoprophylaxis tools. Existing immunization attitude scales were not directly applicable to pediatricians or to the context of RSVpreF and nirsevimab implementation. Consequently, the questionnaire was developed de novo, guided by expert review, pilot testing, and current national and international recommendations. This approach is consistent with other recent KAP studies on RSV prevention among pediatricians, which have also relied on non-validated tools due to the lack of standardized instruments [19–23]. Future research should focus on developing and validating structured tools to assess provider readiness as RSV prevention strategies continue to evolve.

A further limitation relates to the use of dichotomous (yes/no) items to assess attitudes and practices. Although such items enhance clarity and feasibility, they do not allow for the full range of attitudinal gradations typically captured through Likert-type scales. Moreover, binary responses may increase the risk of socially desirable answering, particularly for normatively charged items such as trust in vaccines. The absence of validated Likert-based instruments specific to RSV immunoprophylaxis at the time of study design contributed to this methodological choice. Future research would benefit from the development and validation of multi-item attitude scales tailored to RSV prevention to capture more nuanced provider perspectives.

Finally, the attitude items focused on general vaccination confidence rather than RSV-specific attitudes. This decision reflected the absence of validated instruments tailored to RSVpreF or nirsevimab at the time of study design, as well as evidence that general vaccine confidence is a strong predictor of clinicians’ acceptance of newly introduced immunization tools. Nonetheless, this approach may not fully capture nuances specific to RSV prevention, and future studies would benefit from the development of RSV-specific attitude measures.

The generalizability of these findings should be interpreted with caution. The study was conducted exclusively in Crete, a region with historically high vaccination coverage and a strong pediatric care network, which may not fully represent other areas of Greece with different healthcare structures or immunization cultures. Moreover, differences in RSV prevention policies, provider training, and implementation timelines across European countries may limit the applicability of these results beyond the national context.

Although the study was conducted in a single region, several elements of the local context—such as high baseline vaccination coverage, strong continuity of care between families and pediatricians, and the early introduction of RSV immunoprophylaxis—are shared with other European settings preparing for or undergoing similar implementation processes. Therefore, while the findings should be interpreted within their regional context, they may offer transferable insights for comparable healthcare environments and contribute to broader discussions on provider readiness during the rollout of new RSV prevention strategies [24]. Future studies using nationally representative samples and cross-country comparisons would help clarify whether the patterns observed here reflect broader trends within Greece or across Europe.

Despite these limitations, the study provides timely and actionable insights into the capacity of pediatricians to adopt emerging RSV prevention strategies. The high baseline levels of knowledge and strong vaccine confidence observed among clinicians in Crete suggest that Greece has a strong platform for integrating new immunization tools. At the same time, the specific gaps identified—particularly regarding nirsevimab—highlight the need for targeted educational initiatives as national recommendations evolve. Strengthening dissemination of guidance from the National Immunization Committee, incorporating RSV immunoprophylaxis into continuing medical education, and embedding new preventive tools within existing reimbursement and vaccine delivery structures may facilitate smoother implementation. Ensuring that pediatricians receive timely, practical information on indications, co-administration, and communication with families will be essential for achieving consistent and equitable uptake once RSV preventive measures become routinely available. These findings can support national planning efforts and inform the development of focused training materials to enhance frontline capacity.

## Conclusion

This study provides an early snapshot of pediatricians’ preparedness to incorporate newly available RSV immunoprophylaxis tools into clinical practice in Greece. The consistently high knowledge levels and strong trust in vaccination observed among clinicians suggest that the pediatric care system is well positioned to support the transition to expanded RSV prevention. At the same time, the specific areas of uncertainty identified—particularly regarding the characteristics and indications of nirsevimab—underscore the importance of timely, structured dissemination of evolving recommendations. As national policies continue to develop, ensuring that pediatricians have clear, accessible, and up-to-date guidance will be essential for achieving uniform implementation and maximizing protection for young infants.

## Supplementary Information

Below is the link to the electronic supplementary material.

ESM 1 Supplementary Material 1 (DOCX 19.0 KB)

## Data Availability

Yes. This study is based on primary research data generated through an electronic questionnaire administered to pediatricians in Crete. All data were collected specifically for the purposes of this study.

## Abbreviations

RSV: Respiratory syncytial virus
LRTI: Lower respiratory tract infection
EMA: European Medicines Agency
RSVpreF: Prefusion F Protein RSV Vaccine
NIP: National Immunization Program
KAP: Knowledge, attitudes, and practices
CI: Confidence interval
SD: Standard deviation
