# Supplementary material for: Knowledge, attitudes, and practices of pediatricians in Crete, Greece, regarding RSV immunization: a cross-sectional study
Source: Eur J Pediatr. 2026 Apr 27;185(5):305. doi: 10.1007/s00431-026-06977-5 (PMC13121401; doi:10.1007/s00431-026-06977-5)
Supplement: Supplementary file 1 — Supplementary Material 1 (DOCX 19.0 KB) [file 431_2026_6977_MOESM1_ESM.docx]

**Appendix A. Questionnaire Used in the Study**

**Section 1. Demographic Information**

1. **Age (years)**
2. **Biological sex**
   - 1 = Female
   - 2 = Male
3. **Professional status**
   - 1 = Board‑certified pediatrician
   - 2 = Pediatric resident

**Section 2. Knowledge Assessment**

1. **To what extent do you consider yourself knowledgeable about RSV infection?**
   - No knowledge
   - Moderate knowledge
   - Adequate knowledge
2. **Is there currently an available and safe RSV vaccine for use in the pediatric population?**
   - Yes / No
3. **Do the most recent RSV immunization recommendations include infants up to 6 months of age?**
   - Yes / No
4. **Do the most recent RSV immunization recommendations include children and adolescents?**
   - Yes / No
5. **Do the Greek national guidelines include specific recommendations regarding RSV immunization during pregnancy?**

- Yes / No

1. **Is RSV vaccination recommended for pregnant women according to the latest guidelines of the National Immunization Committee?**

- Yes / No

1. **Can RSV be transmitted through food consumption?**

- Yes / No

1. **Is RSV transmitted through respiratory droplets?**

- Yes / No

1. **Does RSV share common clinical symptoms with influenza?**

- Yes / No

1. **Is there a specific antiviral treatment available for RSV?**

- Yes / No

1. **In Europe, is the RSV season recognized as occurring from November to March?**

- Yes / No

1. **Are ready‑to‑use monoclonal antibodies available for immunoprophylaxis of newborns and infants against RSV?**

- Yes / No

1. **Are there differences between the two available monoclonal antibodies against RSV, Synagis (palivizumab) and Beyfortus (nirsevimab)?**

- Yes / No

1. **Is Synagis (palivizumab) administered to newborns and infants with specific underlying medical conditions?**

- Yes / No

1. **Is apnea one of the most common complications of severe RSV infection in infants under one year of age?**

- Yes / No

1. **Are antibiotics effective in treating RSV infection?**

- Yes / No

1. **Do maternal antibodies against RSV reduce the risk of infection in infants during the first 4–6 months of life?**

- Yes / No

**Section 3. Attitudes and Practices**

**Practice items**

1. **Do you strictly follow the vaccination recommendations of the National Immunization Committee, both personally and for your patients?**
   - Yes / No

**Attitude items**

1. **Do you generally trust vaccines?**
   - Yes / No
2. **Do you consider vaccines essential for controlling infectious diseases and protecting public health?**
   - Yes / No
3. **Do you believe that vaccines significantly reduce morbidity and mortality caused by various diseases?**
   - Yes / No
